# Summers with low Arctic sea ice linked to persistence of spring atmospheric circulation patterns

**DOI:** 10.1007/s00382-018-4279-z

**Published:** 2018-05-30

**Authors:** Marie-Luise Kapsch, Natasa Skific, Rune G. Graversen, Michael Tjernström, Jennifer A. Francis

**Affiliations:** 1 Department of Meteorology and Bolin Centre for Climate Research, Stockholm University, 10691 Stockholm, Sweden; 2 Max-Planck Institute for Meteorology, Bundesstraße 53, 20146 Hamburg, Germany; 3 Department of Marine and Coastal Sciences, Rutgers University, 71 Dudley Road, New Brunswick, NJ 08901 USA; 4 Department of Physics and Technology, University of Tromsø, Postbox 6050, Langnes, 9037 Tromsø, Norway

**Keywords:** Climate variability, Arctic sea ice, Self organizing maps (SOMs), Atmospheric circulation, Atmospheric energy transport

## Abstract

The declining trend of Arctic September sea ice constitutes a significant change in the Arctic climate system. Large year-to-year variations are superimposed on this sea–ice trend, with the largest variability observed in the eastern Arctic Ocean. Knowledge of the processes important for this variability may lead to an improved understanding of seasonal and long-term changes. Previous studies suggest that transport of heat and moisture into the Arctic during spring enhances downward surface longwave radiation, thereby controlling the annual melt onset, setting the stage for the September ice minimum. In agreement with these studies, we find that years with a low September sea–ice concentration (SIC) are characterized by more persistent periods in spring with enhanced energy flux to the surface in forms of net longwave radiation plus turbulent fluxes, compared to years with a high SIC. Two main atmospheric circulation patterns related to these episodes are identified: one resembles the so-called Arctic dipole anomaly that promotes transport of heat and moisture from the North Pacific, whereas the other is characterized by negative geopotential height anomalies over the Arctic, favoring cyclonic flow from Siberia and the Kara Sea into the eastern Arctic Ocean. However, differences between years with low and high September SIC appear not to be due to different spring circulation patterns; instead it is the persistence and intensity of processes associated with these patterns that distinguish the two groups of anomalous years: Years with low September SIC feature episodes that are consistently stronger and more persistent than years with high SIC.

**Electronic supplementary material:**

The online version of this article (10.1007/s00382-018-4279-z) contains supplementary material, which is available to authorized users.

## Introduction

The Arctic sea–ice extent has declined significantly during recent years, with the largest reduction in September (Serreze and Stroeve 2015; IPCC 2013). Superimposed on this trend is a large inter-annual variability; for example, the ice extent in September 2013 and 2014 was about 1.7 million km^2^ higher than the record minimum in 2012, when September sea ice was reduced to 3.6 million km^2^. It has been suggested that about half of the trend can be attributed to rising surface-air temperatures, as a direct result of increasing atmospheric greenhouse gases (Kay et al. 2011; Burt et al. 2016). Other processes contributing to the recent sea–ice trend include indirect effects of global warming and internal variability of the climate system (Kay et al. 2011; Döscher et al. 2014), for example, cloud cover anomalies (Eastman and Warren 2010), variability of ocean currents (Comiso et al. 2008), and shifts and variability of atmospheric circulation patterns (e.g. Overland et al. 2008; Ogi and Wallace 2012; Ding et al. 2017). In order to interpret trends and variability of sea ice it is hence important to also understand the atmospheric and oceanic variability in the Arctic. Here, we focus on the atmospheric variability.

In this study we investigate the atmospheric processes during years with anomalously low summer sea ice since 1979. Recent studies have pointed to the specific importance of atmospheric conditions during spring and early summer for the seasonal sea–ice evolution (Eastman and Warren 2010; Devasthale et al. 2013; Kapsch et al. 2013, 2016; Cox et al. 2016). Using a 1-year observational data set, Persson (2012) found that although melt onset in the Arctic does not occur before May, significant surface warming associated with atmospheric processes that increase the energy flux to the surface can occur months before the melt. Kapsch et al. (2013) showed that positive anomalies of clouds and atmospheric water vapor are present over the part of the Arctic that exhibits the largest sea–ice variability in spring of years with anomalously low September sea–ice concentrations (SICs). In years with relatively high SICs, the opposite situation prevails. Positive anomalies of clouds and water vapor in spring lead to enhanced energy flux to the surface, specifically in the form of more downward longwave radiation (LWD), and an earlier melt onset (Mortin et al. 2016; Lee et al. 2002). These cloud and water–vapor anomalies are linked to anomalous moisture transport into the Arctic, indicating a remote origin (Kapsch et al. 2013; Mortin et al. 2016).

Here, we investigate whether positive anomalies of the energy flux to the surface are associated with certain atmospheric circulation patterns that drive anomalous transport of moisture into the region of largest sea–ice variability (Kapsch et al. 2013). For the energy flux, we specifically focus on the net longwave radiation and turbulent fluxes (LWNT) during spring, prior to melt onset. The circulation patterns are identified based on a Self-Organizing-Maps (SOMs; e.g. Kohonen 1982, 2001; Skific and Francis 2012) algorithm. We seek to answer two major questions: (1) are there specific spring atmospheric circulation regimes that favor atmospheric moisture or heat transport into the Arctic in years with a low September SIC? and (2) are there systematic differences in the character, e.g., duration and strength, of spring atmospheric transport events in years with a low September SIC versus years with a high SIC? In order to approach these questions, we present a characterization of individual events, attempting to provide a step forward in our understanding of important processes governing the large variability of the ice cover.

## Data and methods

### Reanalysis data

All data used in this study come from the ERA-Interim reanalysis by the European Center for Medium-Range Weather Forecast (ECMWF; Dee and Uppala 2009; Dee et al. 2011) for 1979–2012, at a 0.5°x0.5° horizontal grid resolution. Despite known shortcomings, ERA-Interim is acknowledged as one of the best reanalyzes in representing the Arctic climate (e.g., Jakobson et al. 2012; Kapsch et al. 2013—Supplementary Information; Lindsay et al. 2014). We use vertically integrated cloud water, near-surface air temperatures and winds, and geopotential heights averaged daily from the 6-hourly ERA-Interim analyses. Daily averaged SICs are also taken from ERA-Interim; note, however, that SICs are not a model product in ERA-Interim, but are prescribed from independent analyses, based on space-borne sensors (Dee et al. 2011). For radiation fluxes, evaporation, and precipitation we use accumulated values from 24-h ERA-Interim forecasts initiated at 00 UTC.

The atmospheric energy transport is calculated at 6-hourly resolution, based on the model’s hybrid levels and corrected for a mass-flux inconsistency in ERA-Interim (Trenberth 1991; Graversen et al. 2007). The energy transport is separated into its dry-static and latent components:

1 $${\mathbf{J}}_{{{\mathbf{dry}}}} = \frac{1}{{\text{g}}}\mathop \int \limits_{0}^{1} {\mathbf{v}}\left( {\frac{1}{2}{\mathbf{v}}^{2} + {\text{c}}_{p} {\text{T}} + {\text{gz}}} \right)\frac{{\partial {\text{p}}}}{{\partial \eta }}d\eta$$

2 $${\mathbf{J}}_{{{\mathbf{latent}}}} = \frac{1}{{\text{g}}}\mathop \int \limits_{0}^{1} {\mathbf{v}}~{\text{Lq}}\frac{{\partial {\text{p}}}}{{\partial \eta }}d\eta ,$$

where **v**(u,v) is the horizontal wind vector, c_p_ is the specific heat capacity of moist air at constant pressure, T is the temperature, gz is the geopotential height, L is the latent heat of vaporization, q is the specific humidity, and η is the vertical model hybrid coordinate. The transports are vertically integrated over the entire atmospheric column. Convergences of latent (ConLE) and dry-static energy (ConDE) are calculated from horizontal divergence of ${{\mathbf{J}}_{{\mathbf{dry}}~}}$ in Eq. (1) and ${{\mathbf{J}}_{{\mathbf{latent}}}}{\text{~}}$in Eq. (2).

### Determination of years with extreme sea–ice conditions

**Fig. 1:**
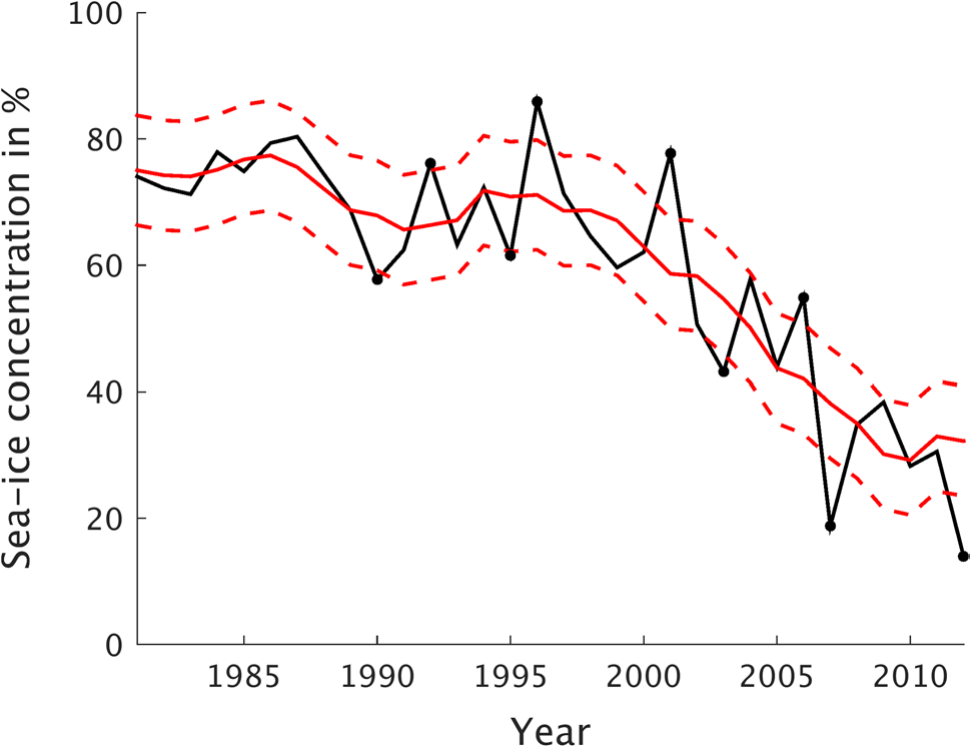
September sea–ice concentration (SIC) in percent for the years 1979–2012. September SICs averaged over the investigation area (black) are shown together with the 5-year running mean of the SIC time series (solid red). Dashed lines indicate where the SICs depart ± 1 standard deviation from the running mean values. Black dots mark years with anomalously low or high September SICs (see Sect. 2.2 for further details). Note that the running mean time series is based on SICs from 1979 to 2014

**Fig. 2:**
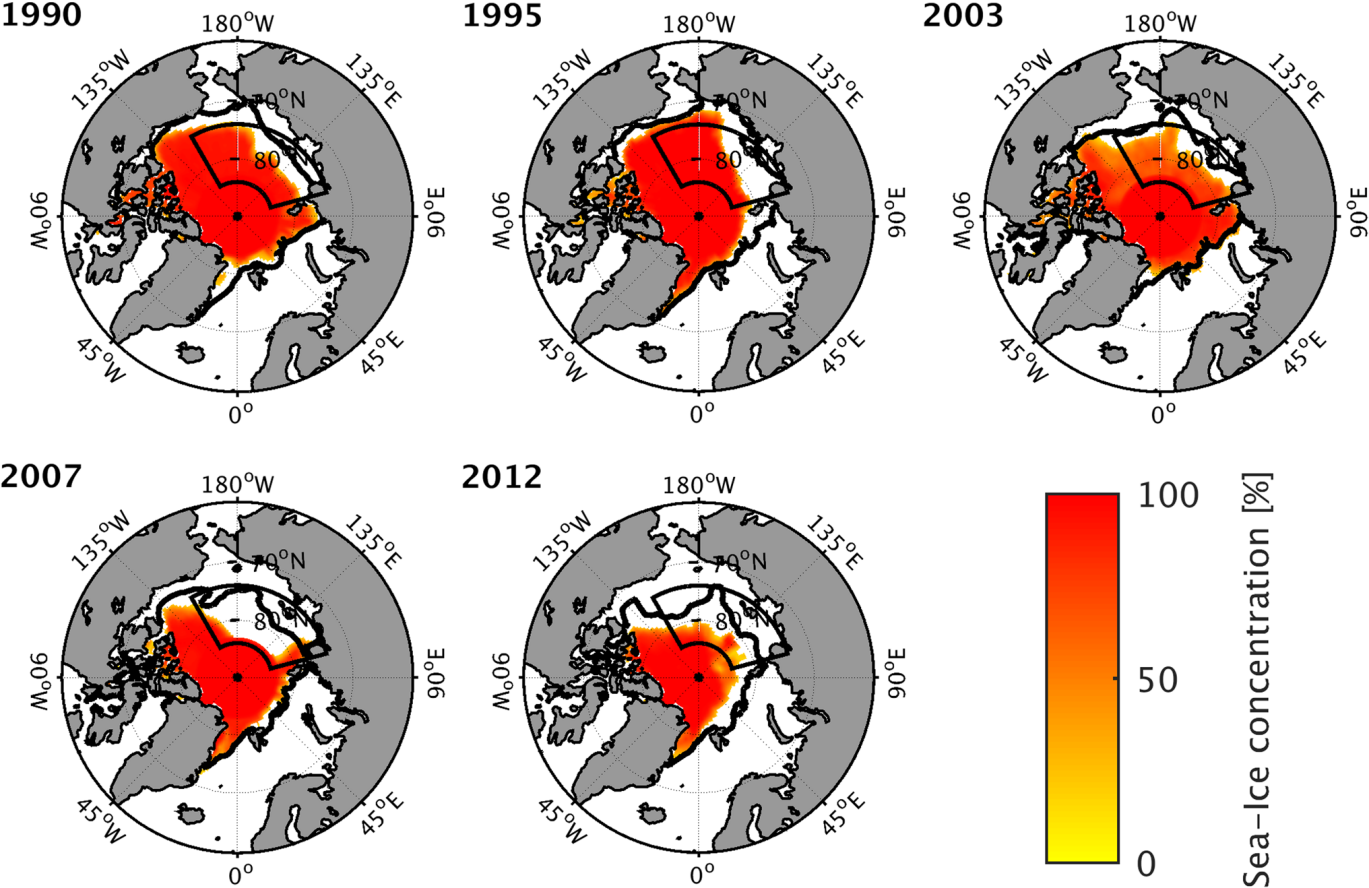
Sea ice conditions during years with an anomalous low September sea–ice concentration (SIC). September SICs (color) and the ice-edge (SIC > 15%) for the respective 5-year climatology (black line) are shown. The black box indicates the investigation area

Figure 1 shows the time evolution of the September SIC for 1979–2012, averaged over the area where September SIC variability is the largest (Fig. 2; Kapsch et al. 2013); this area is shown in Fig. 2, extending from the Beaufort Sea over the East Siberian and Laptev Seas towards the Kara Sea (105°E to 150°W and 74°N to 84°N). This area is hereafter referred to as the investigation area. Because the intention here is to explore the atmospheric dynamics for years with anomalously low summer sea–ice conditions, the SIC variability is separated from its trend. A 5-year running mean over the SICs from 1979 to 2014 provides a reference SIC climatology, yielding the SIC anomalies when subtracted from the original SIC data (Table 1). Note, that in the first and last 2 years of the time series, the 5-year reference climatology is a weighted mean over the adjacent years, constructed such that the anomalies add up to zero over the time series (i.e., $~{\bar {x}_1}=\frac{1}{5}~\left[ {2{x_1}+2{x_2}+{x_3}} \right];\,{\bar {x}_2}=\frac{1}{5}~[2{x_1}+{x_2}+{x_3}+{x_4}]~$).

**Table 1:** Characteristics of episodes with positive longwave net radiation plus turbulent flux anomalies (LWNT), the remaining days during spring (Mar 1 to melt onset, excluding LWNT episodes) and spring of years with a low/high September sea–ice concentration (LIYs/HIYs)

	Year	Number of LWNT episodes	Duration of episodes [days]	LWNT anomaly during episodes of positive LWNT multiplied by duration of events [10^8^ Jm^−2^]	LWNT anomaly during remaining spring days multiplied by # of days [10^8^ Jm^−2^]	LWNT anomaly during spring multiplied by # spring days [10^8^ Jm^—2^]	September sea–ice concentration anomaly [%]
LIYs	1990	**14**	**62**	**40**	− **2**	**48**	− 10.2
	1995	**13**	**56**	**32**	− **9**	**31**	− 9.3
	2003	**9**	32	10	− **7**	**18**	− 11.5
	2007	**9**	**39**	**17**	− 19	**7**	− 19.4
	2012	8	33	8	− 19	− 10	− 18.2
HIYs	1992	**6**	**21**	**5**	− **32**	− **16**	9.8
	1996	8	37	16	− **31**	− 4	14.7
	2001	**5**	**14**	**1**	− **31**	− **29**	19.1
	2006	**6**	34	10	− **18**	− 5	20.1
All years^a^	1979–2012	8	34	10	− 19	− 3	− 0.53

**Fig. 3:**
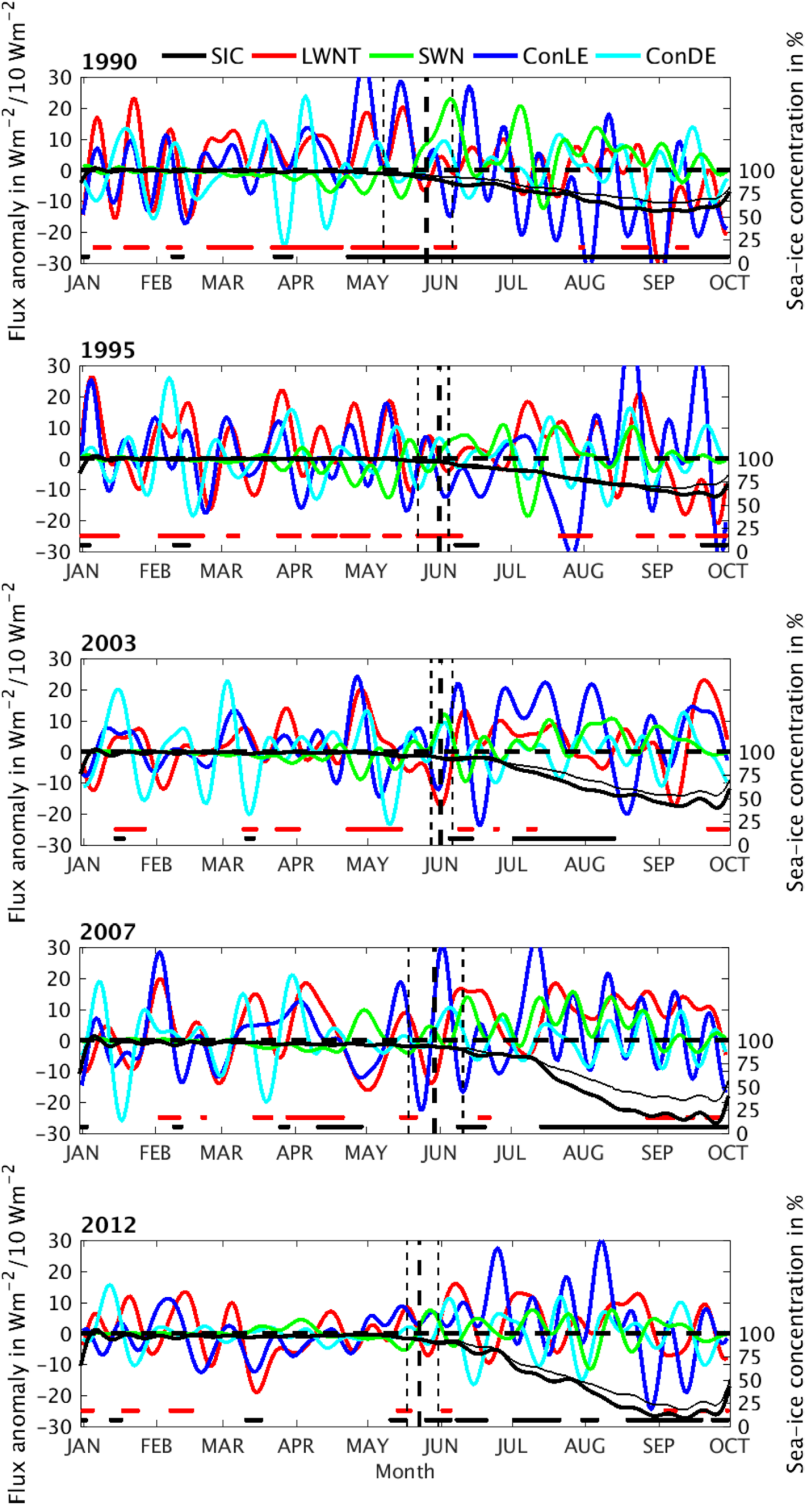
Atmospheric variables and anomalies for the five LIYs. Shown are anomalies of positive net longwave radiation plus turbulent fluxes (LWNT; red), net shortwave radiation (SWN; green) and convergence of latent (ConLE; blue) and dry-static energy (ConDE; cyan). The flux anomalies are positive if downward to the surface. SICs are shown for the respective year (thick black) together with the 5-year climatology (thin black). Days with significantly larger LWNT anomalies and smaller SICs are indicated with horizontal lines (α = 0.05; red and black, respectively). Units for LWNT and SWN are in Wm^−2^, for ConQE and ConDE in 10^−1^ Wm^−2^ and for SICs in %. A 14-day low-pass fft-filter is applied to all time series for visualization. Vertical lines indicate the begin of melt onset (BMO), median melt onset (MMO), and end of melt onset (EMO; see Table 2; Sect. 2.4), respectively

All values are averages over the area indicated by the black box in Fig. 2. The last row shows the characteristics for all years between 1979 and 2012; note, the values are normalized by the number of years included in the time period. The last column presents the values for the Northern Hemisphere sea–ice concentration anomalies as shown in Fig. 3

Values in bold indicate statistical significance. A one-sided student’s t test was applied (α = 0.05), with the null-hypothesis that individual values are larger (LIYs) or smaller (HIYs) compared to the distribution of all years

^a^The values are normalized by the number of years

**Table 2:** Melt dates for years with low (LIY) and high (HIY) September sea–ice concentrations

	Years	BMO [DOY]	MMO [DOY]	EMO [DOY]
LIYs	1990	128** (144)	146** (155)	157** (164)
	1995	143** (146)	152** (155)	156** (164)
	2003	148* (146)	152 (152)	157** (158)
	2007	139 (140)	150 (149)	162* (157)
	2012	138** (141)	143** (149)	151** (154)
HIYs	1992	152* (144)	161* (154)	176* (164)
	1996	144** (147)	160* (156)	166* (163)
	2001	151* (147)	159* (154)	162* (159)
	2006	136** (141)	143** (150)	153** (159)

Melt dates are averaged over the area specified in Fig. 2. Shown are the days of the year (DOY) for the median melt onset (MMO; see Sect. 2.4) as well as the begin of melt onset (BMO) and end of melt onset (EMO). BMO is defined as the date on which melt onset has occurred in all grid boxes of the area, and EMO as the date on which melt onset has occurred in all grid boxes of the area. Please refer to Sect. 2.4 for details

Statistically significant earlier (**) and later (*) melt onset; values in parenthesis display respective 5-year climatology

Applying a 5-year running mean as reference climatology allows for an investigation of the year-to-year variability without a priori specification of the characteristics of the underlying trend (e.g., whether it is linear or of higher order). Years with an anomalously low (high) SIC are determined as years that fall below (above) one standard deviation of the residual anomaly time series. This results in five years with anomalously low (hereafter referred to as LIYs) and 4 years with anomalously high SICs (similarly referred to as HIYs; Fig. 1). Tests with a somewhat longer averaging period than 5 years for the reference climatology change the classification only slightly; the most extreme LIYs are identical. The five identified LIYs are 1990, 1995, 2003, 2007 and 2012 (Fig. 1); Fig. 2 shows the September SIC for each of these. The four HIYs are 1992, 1996, 2001 and 2006.

### Episodes of an anomalous surface energy budget

Anomalies of each variable considered in the surface-energy budget as well as the energy transports are obtained by removing the annual cycle. The annual cycle is calculated for each grid point using daily resolution data. First, we calculate a 5-year average for each day of a given year, using the same method as applied for the SIC (Sect. 2.2). Second, a 30-day running mean is applied on the annual cycle of each year to suppress inter-monthly variability. The anomalies are then obtained as the residual of the actual time series and the annual cycle climatology. Finally, the anomaly (the residual) is averaged over the investigation area. The reanalysis data used for this study comprise the years 1979–2012. The final time series of the anomalies for all the identified LIYs and HIYs are displayed in Fig. 3 and Figure S1 in the Supplementary Material, respectively.

We only consider episodes of positive LWNT anomalies between the first of March and melt onset, defined below. If several individual episodes appear, such as for example in late March and early April of 1990 (Fig. 3), each episode is treated individually.

Statistical significance of anomalies in LWNT and other variables presented in Fig. 3 and S1 are determined using a one-sided student’s *t* test (α = 0.05), with the null-hypothesis that the anomalies are not significantly different from the respective climatology, unless stated otherwise.

### Melt onset calculation

**Fig. 4:**
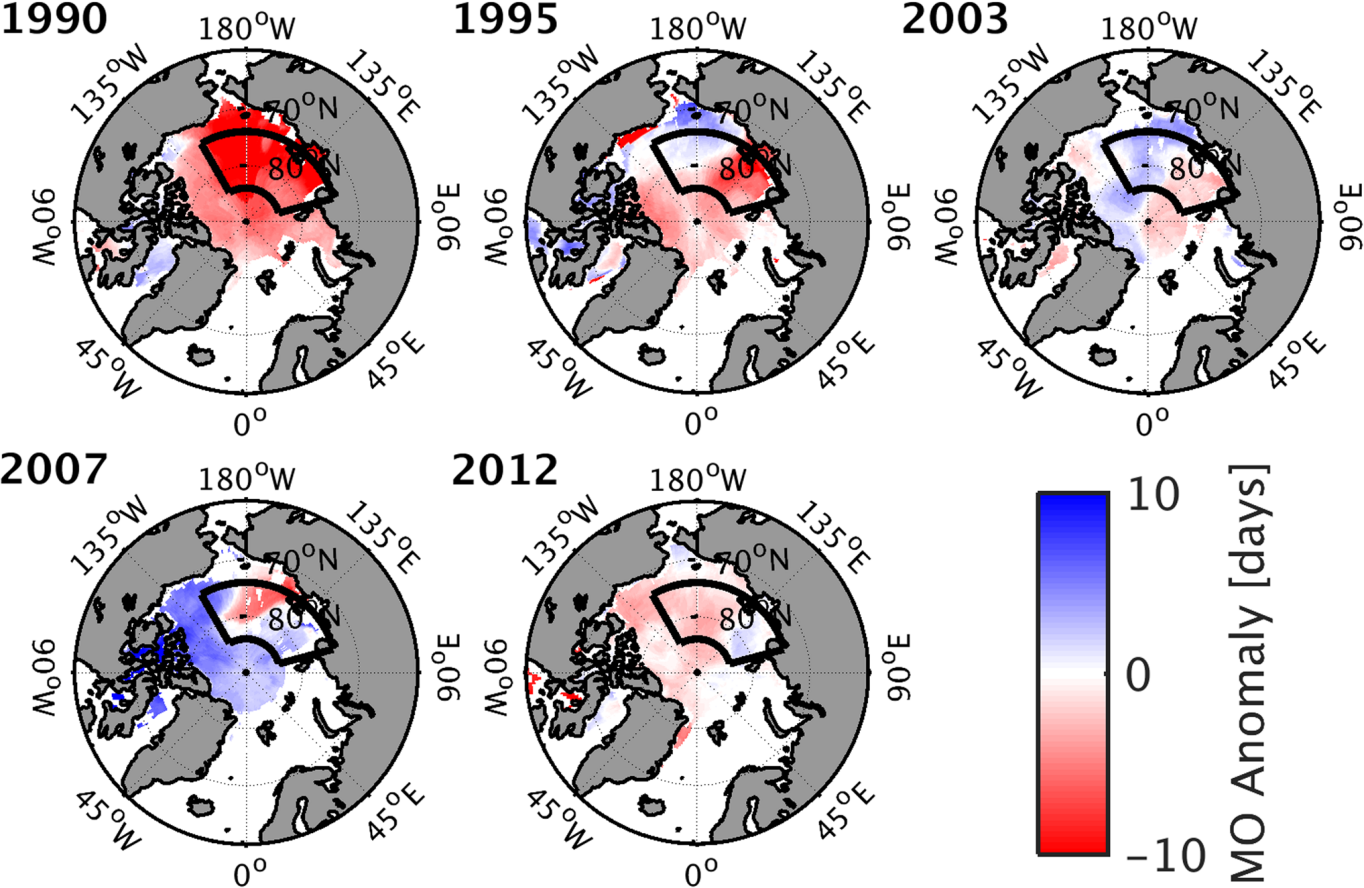
Melt onset date anomalies during LIYs. The black box indicates the investigation area (see Sect. 2.4 for details)

The melt onset dates for LIYs and HIYs are given in Table 2. The melt onset is determined as the first day in spring when the 14-day running median of the near-surface air temperature exceeds − 1 °C (Rigor et al. 2002; Persson 2012). Melt onset is calculated for each grid point with more than 80% sea ice for at least 98% of the days in January through April (for details see Mortin et al. 2014). In the following we define the date of melt onset over the investigation area as the date when half of the grid boxes in the area have experienced melt onset, hereafter referred to as median melt onset (Table 2). The spatial distribution of the melt onset date anomaly for each of the LIYs is displayed in Fig. 4. The anomalies for each year and grid point are relative to a 5-year reference climatology, as described in Sect. 2.2.

### Self-organizing maps (SOMs)

To identify atmospheric circulation patterns, a Self-Organizing Map (SOM) algorithm is used. The process of SOM formation starts by creating an initial set of reference vectors that best describes a 3-dimensional data sample. In the training stage, these reference vectors are created by placing the first and second empirical orthogonal function (EOF)-based vectors in the diagonally opposite corners of the map matrix, then interpolating the remaining space between them. During the training, the vectors adjust depending on the Euclidean distance from the data sample; the closer the vectors are to the data sample, the more they are adjusted. The training is complete once the set of vectors is found that minimizes the distances and best describes the given data. An important feature of SOMs is that the initialization of a map, using EOFs, does not precondition its final form, but rather speeds up the training process compared with random initialization. Moreover, one advantages of SOMs over EOFs is that the process of training allows for non-linear features of the complex climate system to be captured, thus providing a more detailed and more realistic view of the data’s true nature (Reusch et al. 2005). More details on the map training can be found in Skific et al. (2009), Skific and Francis (2012) and Hewitson and Crane (2002).

The representation of reference vectors on a 2-dimensional array according to their similarity is a great advantage in atmospheric sciences, because to an experienced meteorologist, this representation is reminiscent of familiar atmospheric circulation features. The interpretation of the SOMs then becomes intuitive as well as quantitative, as one can observe dominant features or transitions between various states (Hewitson and Crane 2002). These unique aspects of SOMs help to explain climate variability and reveal several inherent properties of the climate system: first, they provide structures that are coherent, not a set of discrete unrelated patterns (Sheridan and Lee 2011); second, they reveal structures that can also be highly complex and non-linear.

In this study, the SOM training and analysis are based on daily spatial anomalies of 850 hPa geopotential heights during spring (defined as March 1 to melt onset) of the years 1979–2012. The use of geopotential anomalies, rather than absolute values, ensures that each SOM cluster represents purely circulation-related features rather than features tied to the absolute magnitude of the geopotential. The daily anomalies are calculated by subtracting the geopotential at each grid point from the domain-averaged geopotential for each daily field. Before the SOM training process, geopotential height anomalies are re-gridded from the original 0.5° × 0.5° to a 250 km × 250 km Equal-Area Scalable Earth Grid (EASE). Interpolation to an equal-area grid ensures equal weighting of the grid boxes in the SOM training. Also, it reduces the size of the input data, which speeds up the processing. Further, all grid points over Greenland are removed, as the high topography leads to permanent spatial anomalies in the geopotential fields often unrelated to the dynamic anomalies in the flow field, thereby creating unrealistic features in the SOMs. The resulting set of vectors is displayed in a SOM matrix (hereafter referred to as master SOM; see Skific and Francis 2012), defined as a 12-cluster matrix in order to minimize the size of the matrix while still capturing the essential variability of the data set.

**Fig. 5:**
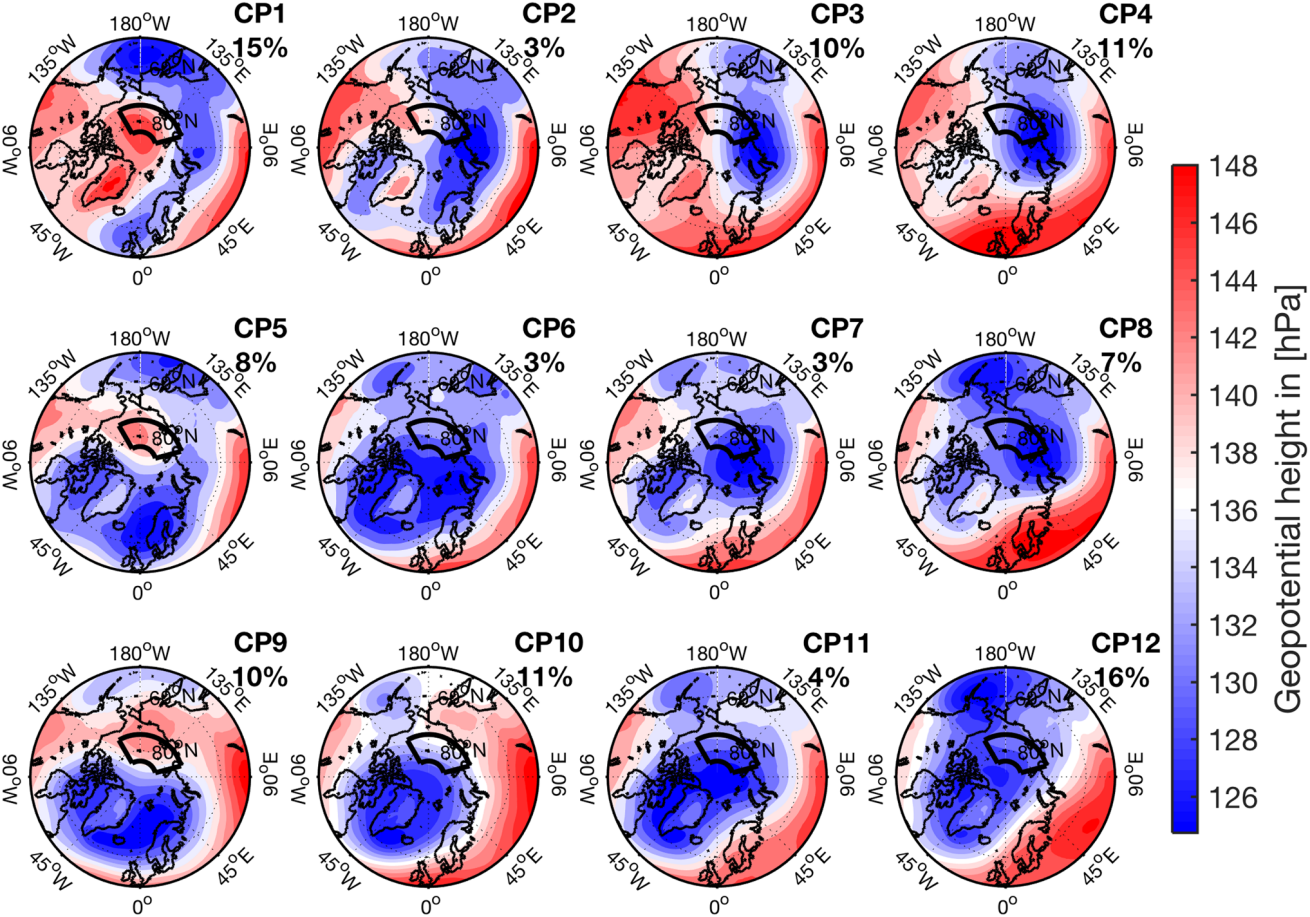
Circulation patterns during spring. Shown are 850 hPa geopotential heights. The patterns are clustered and organized according to the Self-Organizing Map algorithm applied on geopotential height anomalies during spring (March 1 to melt onset) of the years 1979–2012 (see Sect. 2.5). The number on the top right of each map gives the relative frequency each of these patterns occurred throughout 1979–2012 (3240 days in total)

In order to investigate the atmospheric conditions during episodes of enhanced LWNT, atmospheric variables are “mapped onto” each cluster in the master SOM. For the mapping, each day of the investigation period is assigned to the best-match SOM cluster, determined by finding the SOM pattern with the smallest Euclidean distance of the 850 hPa geopotential height anomaly field. Geopotential height fields (not anomalies) during spring of all years are mapped onto the master SOM and displayed in Fig. 5 to characterize each circulation pattern. Other mapped variables include anomalies of latent and dry-static energy transport and convergence, cloud cover, radiation and winds. This feature of SOM analysis allows for a thorough exploration of the characteristics of each cluster (Skific and Francis 2012). Further, atmospheric variables used to calculate LWNT episodes during LIYs and HIYs are mapped onto the SOM clusters individually to identify differences and similarities between the episodes in the respective years.

## Results

### Sea–ice conditions during years of low September sea ice

Except for 2003, the September SIC for the entire Northern Hemisphere is anomalously low during all LIYs, which are identified with respect to the investigation area (Fig. 2). This indicates that the investigation area captures a large part of the Arctic-wide interannual sea–ice variability. Most of the SIC anomalies during LIYs are centered in the Laptev, East Siberian, Chukchi, and Beaufort Seas. During LIYs, the September SIC anomalies in the investigation area are between − 9 and − 19% (Fig. 1; Table 1). Note that owing to the trend of SIC in recent years (e.g., Serreze and Stroeve 2015), the 5-year reference climatology of SIC for 2007 and 2012 is significantly reduced relative to earlier periods along the coasts of the East Siberian, Chukchi and Beaufort Seas (Fig. 2). For example, in September 2007 most of the East Siberian Sea and parts of the Beaufort Sea were ice-free. Yet SIC anomalies in the investigation area still fall more than 18% below the 5-year reference values for 2007 and 2012 (Table 1).

All LIYs exhibit an earlier melt onset than the 5-year reference climatology over the investigation area or parts thereof (Fig. 4). Earlier melt onset over the investigation area is most significant in 1990, occurring on average about 9 days earlier (Table 2). In 2003 and 2007 the area-averaged melt onset is not significantly different from the 5-year mean climatology. In contrast, for HIYs the September SIC anomalies are 10–20% higher than the respective 5-year reference climatology over the investigation area (Table 1; Fig. 1). Also, the melt onset is significantly later for all HIYs except for 2006 (Table 2). Hence these results indicate that the melt onset plays a role for the September sea–ice conditions.

### Spring atmospheric conditions during years of low September sea ice

As displayed in Fig. 3, spring and early summer of LIYs are often characterized by episodes with anomalously positive LWNT over the investigation area. In the five LIYs, between 8 and 14 episodes with enhanced LWNT occur, compared to 5 and 8 for HIYs; the average over all years is 8 (Table 1). The length of the individual events varies between 1 and 7 days (not shown). The LWNT episodes in LIYs are associated with an average LWNT anomaly of approximately 2 GJm^−2^ during a total of 5–9 weeks throughout the entire spring period, compared to less than 1 GJm^−2^ for HIYs during 2–4 weeks. During this time of the year, LWNT anomalies dominate the surface energy budget, as anomalies of shortwave net radiation (SWN) contribute little to the surface energy budget owing to a high surface albedo and, in the beginning of the season, low solar inclination. Changes in LWNT are associated with variations in surface and near-surface air temperature, water–vapor content, and cloud cover. Variations in water vapor and clouds alter the emissivity of the atmosphere and hence LWD at the surface.

Fewer LWNT episodes tend to occur over the LIYs during the latter part of these years; the number and duration of LWNT episodes are statistically different from the 1979–2012 average only for the years 1990, 1995 and 2007 (Table 1). One factor contributing to this decrease could be related to a shortening of the spring season owing to a significantly earlier melt onset (Stroeve et al. 2014). Further, the LWNT anomalies during the LWNT episodes become weaker with time, while sea–ice concentration anomalies become larger. This is consistent with an Arctic regime shift towards thin first-year ice. Thinner ice melts more easily and is more prone to wind forcing, hence less energy is needed to cause significant ice anomalies. Moreover, as the ice thins and the SIC declines, surface temperatures increase, which leads to an increased upward longwave flux and reduced positive net longwave radiation.

**Fig. 6:**
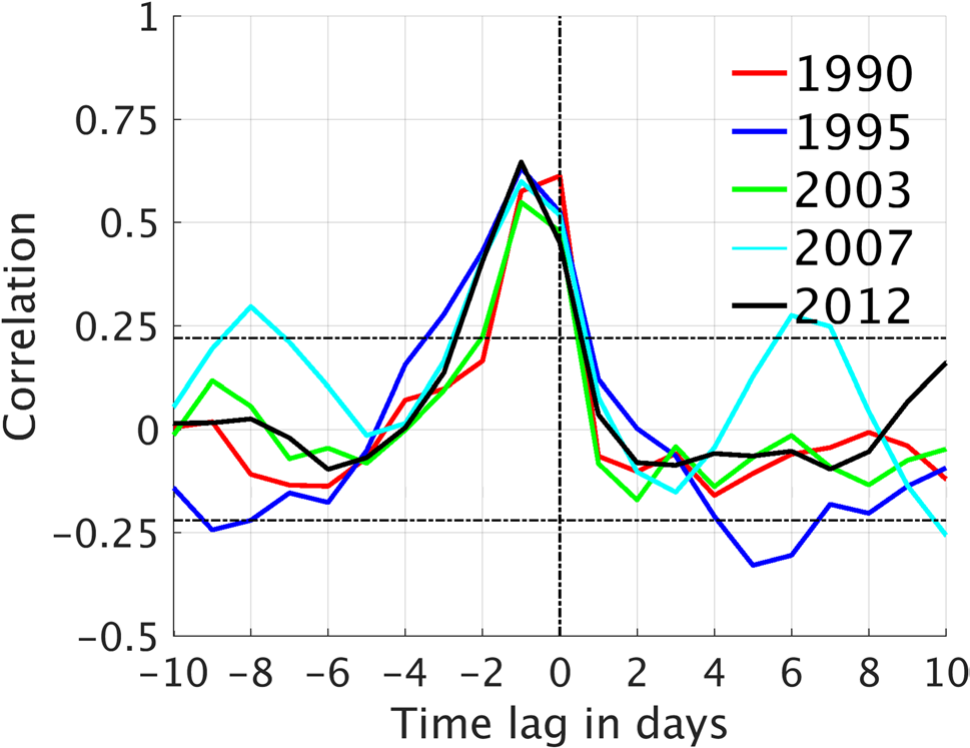
Correlations between anomalies of the net longwave radiation plus turbulent fluxes (LWNT) and the latent (solid) energy convergence as function of time lag. The correlations are calculated for the spring period (March 1 to melt onset) after averaging over the investigation area (see Fig. 2). Values above/below the horizontal lines (black, dashed) are statistically significant correlations (α = 0.05)

Figure 6 shows the lag correlation between anomalies in LWNT and the convergence of moisture advection (ConLE). During LIYs, LWNT anomalies occur in coherence with significant positive anomalies of ConLE, although with a lag in the order of one day; the estimated lag is sensitive to the length of the anomalies. Extended episodes of positive anomalies of ConDE also prevail throughout spring of LIYs, but are less correlated to the LWNT anomalies in most of the years (not shown). This suggests that anomalies of LWNT are mostly affected by anomalies of ConLE, rather than by ConDE. Only 2007 displays a significant correlation between LWNT and ConDE; 0.35 for a 3-day lag (not shown), indicating that ConDE anomalies played a larger role in the surface-energy budget during spring of 2007 than in other LIYs. In 2007, however, an early melt onset occurred only in a small part of the investigation area (Fig. 4), suggesting that melt-related processes during spring contributed less to the 2007 September sea–ice minimum than did other processes. This is consistent with findings by Graversen et al. (2011), who suggested that latent and dry-static energy transports into the Arctic during summer (June to August), rather than in spring, contributed to the sea–ice anomaly in 2007. Zhang et al. (2008) and Kauker et al. (2009) argued that ice dynamics may also have been important. Kauker et al. (2009) found that a large part of the sea–ice reduction in 2007 can be attributed to an anomalously low ice-thickness in March, increased wind stress on ice in May and June, as well as relatively warm sea-surface temperatures throughout September. A warming of the sea–ice layer during spring owing to increased LWNT might, however, have pre-conditioned the ice and altered its sensitivity to other atmospheric and oceanic forcing later in the year.

### Atmospheric circulation associated with LWNT episodes during LIYs

**Fig. 7:**
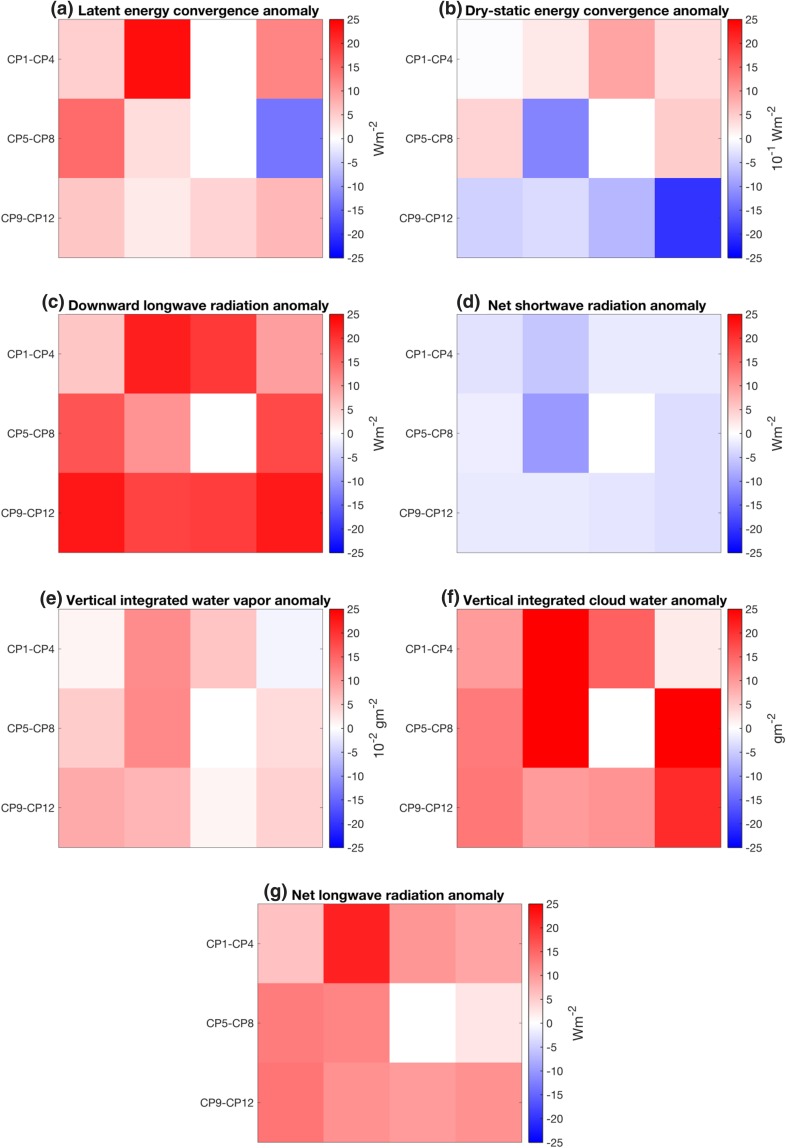
Anomalies of (**a**) latent and (**b**) dry-static energy convergence, (**c**) downward longwave radiation, net (**d**) shortwave radiation, (**e**) cloud water and (**f**) water vapor and (**g**) net longwave radiation during positive LWNT episodes in spring of LIYs for the circulation patterns presented in Fig. 5. Values are averaged over the area indicated by the black box in Fig. 5

**Fig. 8:**
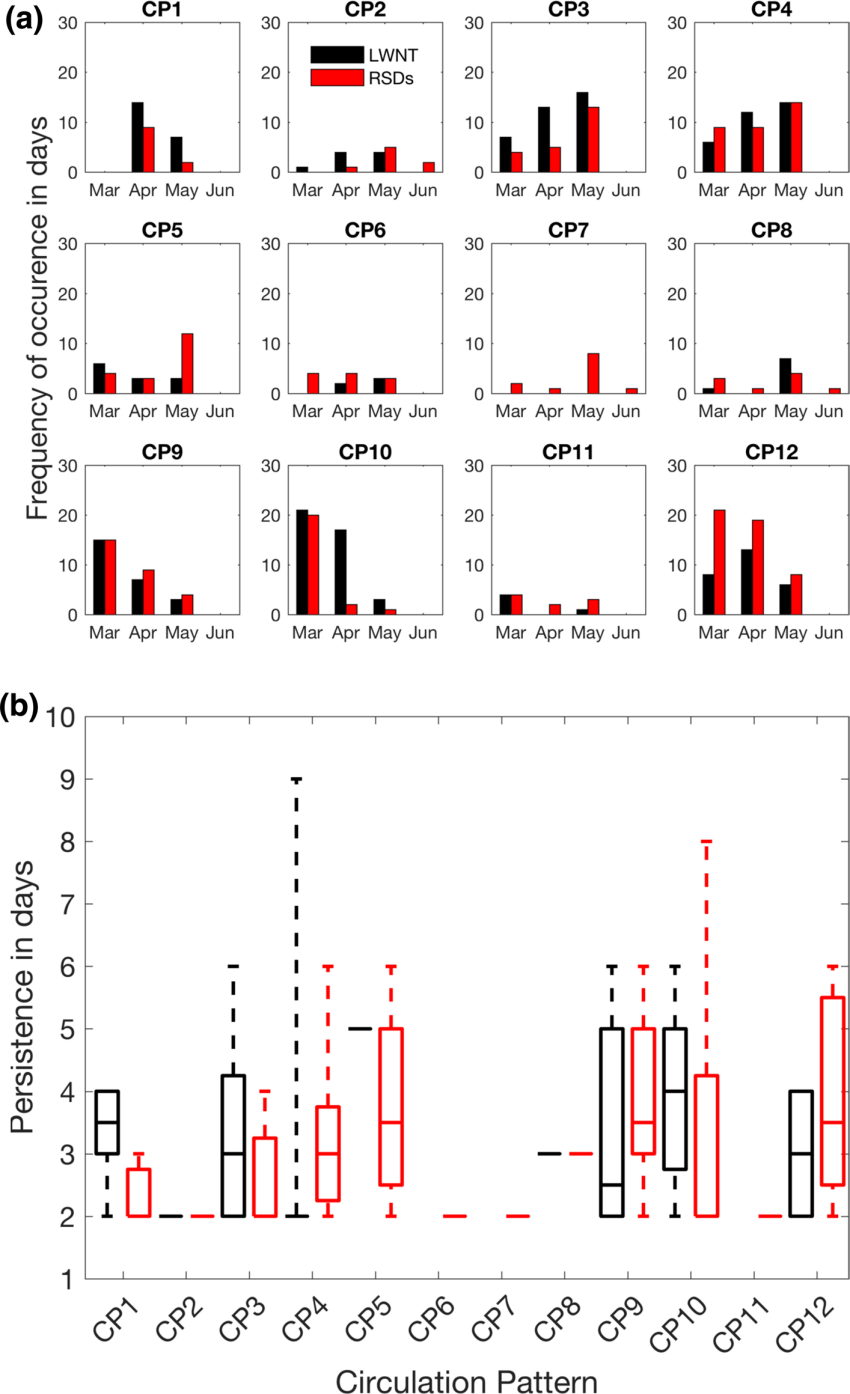
Frequencies of occurrence and persistence of the circulation regimes presented in Fig. 5 during LIYs. **Top**: Frequencies of occurrence during episodes of enhanced longwave net radiation plus turbulent fluxes (LWNT; black) and remaining spring days (RSDs; red) as function of month. Bottom: Persistence of each pattern for LWNT (black) and RSDs (red) of LIYs. Horizontal lines indicate the median persistence, the edges of the boxes the 25th and 75th percentiles and whiskers the 5th and 95th percentiles

In this section the characteristic atmospheric circulation patterns (CPs) during episodes of enhanced LWNT during LIYs are explored. The objective is to ascertain whether common atmospheric conditions prevailed during these episodes of positive LWNT anomalies. Several distinct CPs prevail; we refer to these patterns according to their position in the SOM matrix (CP1–CP12; Fig. 5). To specifically focus on the circulation patterns during episodes of positive LWNT anomaly over the investigation area during LIYs, we map potential contributors to LWNT anomalies onto the master SOM. These are the surface energy fluxes, the convergence of moisture and heat, and the anomalies of integrated cloud water and atmospheric moisture. We do this only for spring days in LIYs with a positive LWNT anomaly over the investigation area; see Fig. 7. Note that by this construction, anomalies in LWD and LWN are positive for all CPs (Fig. 7c, g). Also, CP7 did not occur throughout spring of any of the LIYs. In Fig. 8 we also explore the frequency of occurrence and the persistence of the CPs; only the most frequently occurring CPs will be discussed.

Approximately 44% of the days during LWNT episodes in LIYs are associated with low geopotential heights centered near the Russian coast with high values over the North American side of the Arctic (CP1–CP4; Fig. 5). Such a pattern is often referred to as the Arctic dipole anomaly (AD; Overland and Wang 2005). The AD favors flow from the North Pacific across the central Arctic towards the Atlantic and is associated with northward transport of heat and moisture into the investigation area, causing ConLE and ConDE anomalies to become positive (Fig. 7a, b). This leads to an increase of clouds and anomalously high surface temperatures (not shown) over the investigation area, thereby creating positive anomalies of LWD and LWN at the surface (Fig. 7c, f, g). Note, however, that certain variations in the AD evident in CP1–CP4 may transport air from the cold Asian continent rather than the warm North Pacific into the investigation area, leading to negative surface flux anomalies.

For clusters CP5–CP12 geopotential heights are also low along the Russian coast but, unlike AD patterns, the low geopotential heights extend far west across the Norwegian Sea, Fram Strait, Greenland, and the Canadian archipelago (Fig. 5). The detailed structure differs between these CPs, as the spatial extent of the geopotential heights varies. The associated circulation of most of these patterns results in a westerly flow into the investigation area, with a southwesterly component in patterns with low geopotential heights that extend farther inland over Russia (Fig. 5). The westerly flow brings relatively moist air from Russia and the Kara Sea into the western part of the investigation area, where it leads to positive anomalies in ConLE (Fig. 7a). Only for CP8 are the ConLE anomalies negative over the investigation area, while ConDE anomalies are very small or negative for most of these patterns (Fig. 7b). The fact that almost all patterns during LWNT episodes during LIYs are associated with positive ConLE anomalies supports earlier findings suggesting that moisture convergence triggers positive LWNT anomalies (see Sect. 3.2).

Figure 8 shows the timing in spring of the different patterns (Fig. 8a) and their persistence (Fig. 8b). Note that CP9 and CP10 occur relatively early in spring of LIYs, likely causing an earlier preconditioning of the surface (Fig. 8a). The most persistent circulation patterns during LWNT episodes in spring of LIYs are CP1, CP3, CP5, and CP10 (Fig. 8b). Their median persistence exceeds 3 consecutive days and most of these patterns can last more than 5 consecutive days, giving them a longer time to act on the surface. Consequently, their impact is likely larger than for the other patterns. In contrast to these relatively persistent patterns, CP2, CP6, CP7 and CP11 are transition patterns that occur only rarely during LIYs, and when they do occur they are less persistent than other patterns.

While similar circulation patterns do occur during LWNT episodes and during the remaining spring days (hereafter RSDs; days that fall outside the LWNT episodes in spring of LIYs), there are significant differences in frequencies and persistence of the patterns (Fig. 8). Specifically, CP1, CP3 and CP10 occur more frequently during LWNT episodes than during RSDs of LIYs; 45 and 24%, respectively. These patterns are also more persistent during LWNT episodes than during the RSDs (Fig. 8b). In contrast, CP5 and CP12 occur less frequently during LWNT episodes than during RSDs; 17 and 29%, respectively. Note, that ConLE and ConDE anomalies are very small or negative for CP9 and CP12, which suggests that the positive anomalies of LWNT that are assigned to these CPs are not associated with the convergence of moisture and dry-static energy. Further, almost all patterns show larger positive anomalies of clouds, water vapor, and ConLE during LWNT episodes than during the RSDs (Fig. S2 in the Supplementary Material), indicating that the same patterns have a different impact on the surface during the RSDs.

### Differences in the atmospheric circulation between LIYs and HIYs

**Fig. 9:**
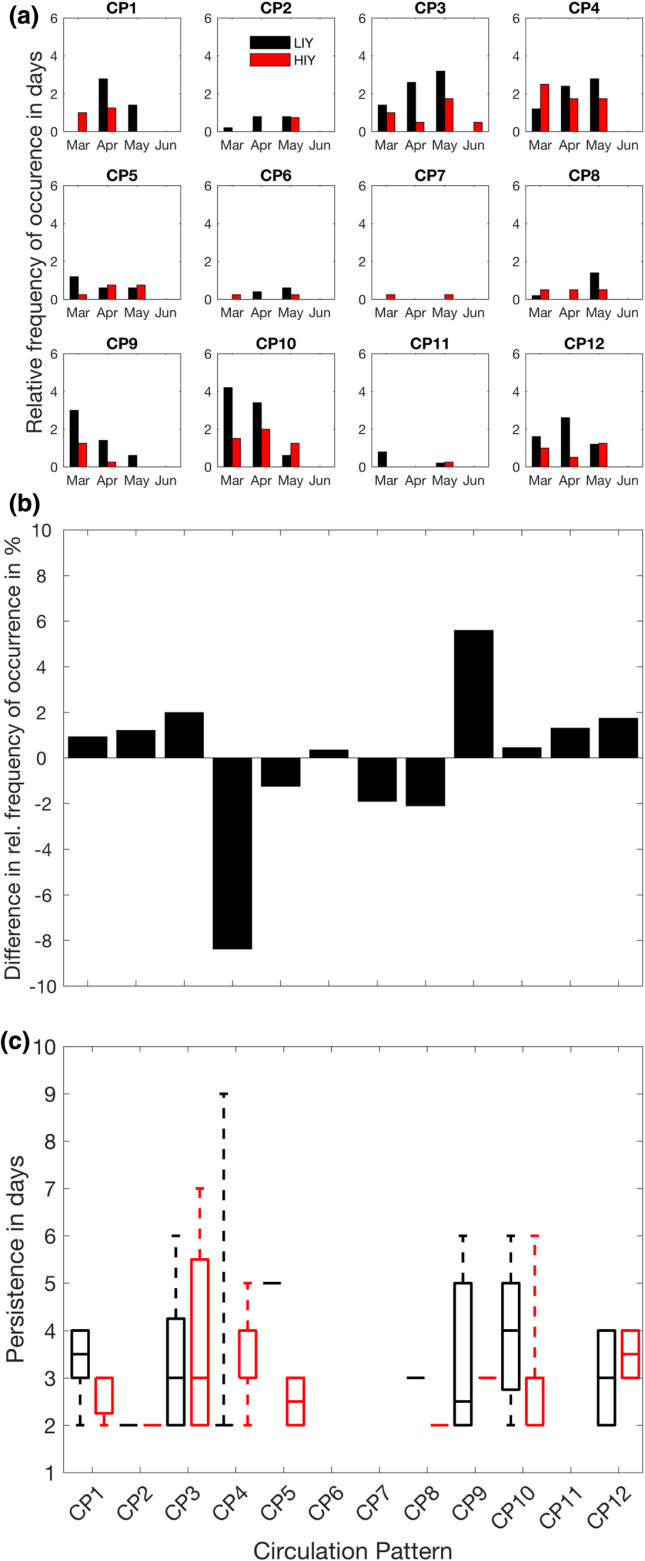
Relative frequency of occurrence of the circulation patterns associated with LWNT episodes during LIYs and HIYs, the difference in the relative frequency between the two data sets as well as the persistence, as presented in Fig. 8b. Top: Relative frequency of occurrence during LWNT episodes of LIYs (black) and HIYs (red) as function of month. The relative frequency is defined as the absolute frequency divided by the total number of days comprising LWNT episodes in LIYs and HIYs, respectively. Middle: Differences in the relative frequencies. Bottom: Persistence for LWNT episodes in LIYs (black) and HIYs (red)

Differences in SIC, melt onset, and frequency and intensity of the LWNT episodes are evident for the LIYs and HIYs (Figs. 3, 9, and S1). The five LIYs are associated with 8–14 episodes of positive surface LWNT anomalies, resulting in an average energy surplus 0.8–4.0 GJm^−2^ over 32–62 days during spring (Table 1). In contrast, HIYs are characterized by fewer and shorter positive LWNT episodes. The four identified HIYs exhibit between 5 and 8 events that are associated with a smaller energy surplus of 0.1–1.6 GJm^−2^ and that span fewer days (20–49 days) as compared to LIYs.

Further, differences in the frequencies and timing of the atmospheric circulation patterns between the two sets of years are found for the LWNT episodes in spring (see Fig. 9). Interestingly, LWNT episodes during LIYs are often characterized by less frequent occurrence of CP4 (an AD pattern) as compared to the HIY LWNT episodes, while CP9–CP12 occur more frequently (Fig. 9a, b). We find that AD-like circulation patterns (CP1–CP4) prevail on about 44% of the LWNT episodes in LIYs and 49% of the days in HIYs (Fig. 9). CP9 to CP12 occur during 44 and 35% of the LWNT episodes for LIYs and HIYs, respectively. The largest differences in frequencies are evident for CP4 and CP9 (Fig. 9b).

Another characteristic feature of the spring circulation patterns in LIYs is the total duration of all LWNT episodes. Figure 9 shows that CP1, CP5, CP8, and CP10 have episodes of positive LWNT anomalies persisting longer in LIYs than for HIYs, with differences becoming more significant for the longer-lasting episodes for all of these patterns except CP10 (Fig. S3 in the Supplementary Material). Three of these patterns also occur earlier in spring, resulting in an earlier preconditioning of the surface in LIYs and, hence, an earlier melt onset (Fig. 4; Tables 1, 2). Owing to the earlier melt onset, feedback mechanisms have longer time to act on the surface. Note that an earlier melt onset, leading to increased absorption of solar radiation throughout the spring and summer season, was found to be one of the major drivers of differences in annual ice evolution (e.g., Wang et al. 2016).

**Fig. 10:**
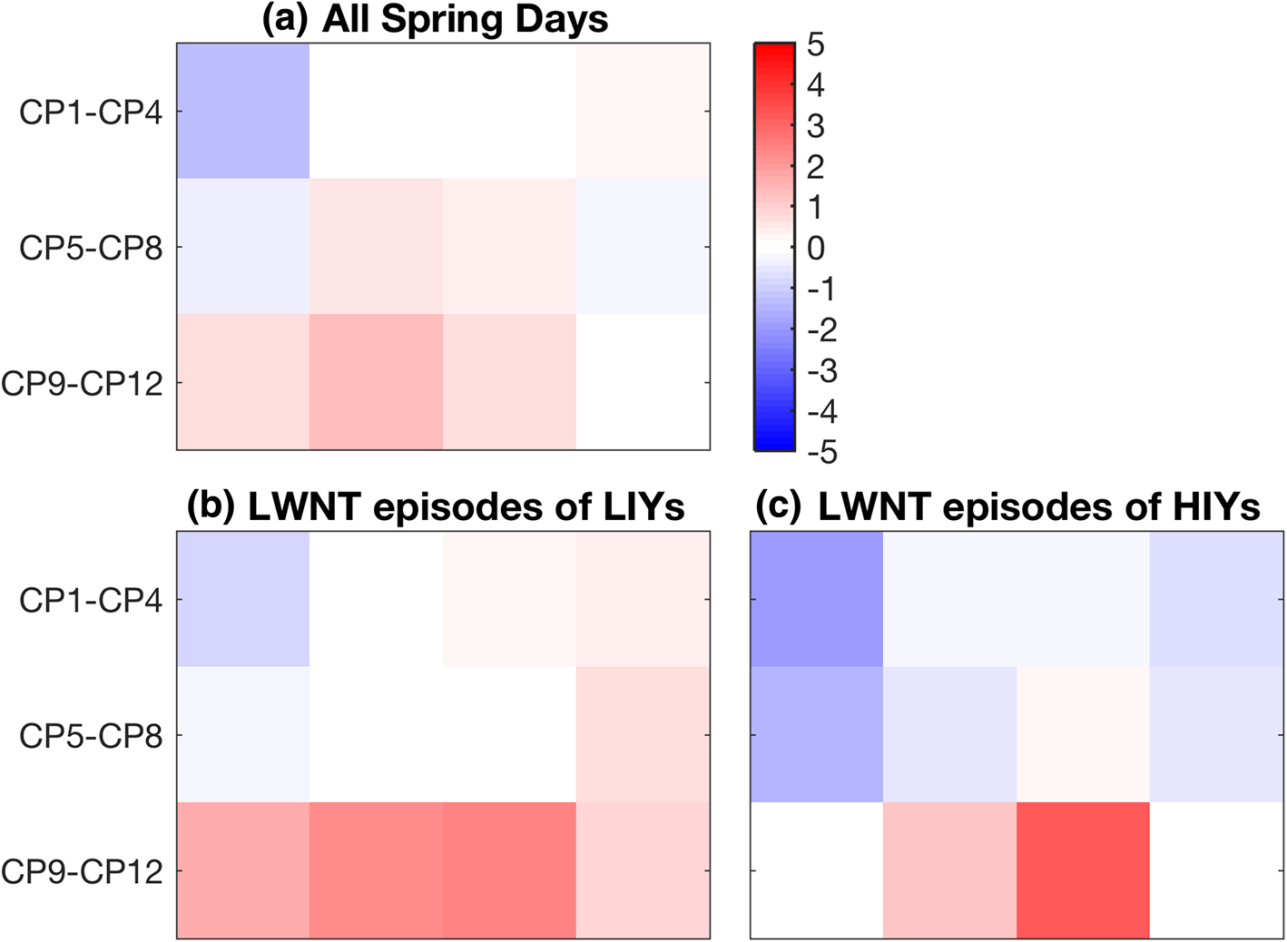
Arctic Oscillation (AO) index mapped onto the circulation regimes of the master SOM. Left: AO index for (**a**) all spring days between 1979 and 2012, and LWNT episodes during springs of years with (**b**) low and (**c**) high September sea–ice concentration (LIYs/HIYs). The AO index was downloaded from NOAA Center for Weather and Climate Prediction, Climate Prediction Center (http://www.cpc.ncep.noaa.gov/products/precip/CWlink/daily_ao_index/ao.shtml)

We also explore possible associations between the occurrence of the LWNT episodes and differing phases of the Arctic Oscillation (AO) by mapping the AO-index onto the SOM matrix in Fig. 10. The AO index is defined as the leading principal component of the Northern Hemisphere sea-level pressure (Thompson and Wallace 1998). A positive index (AO+) represents lower-than-average sea-level pressure over the Arctic. We find that CP4, CP6, CP7 and CP9-CP11 are associated with a positive phase of the AO during spring (Fig. 10a). During LWNT episodes in spring of LIYs, 7 out of the 11 occurring circulation patterns are associated with AO+ (CP3, CP4 and CP8 CP12; Fig. 10b); during LWNT episodes in spring of HIYs only 3 patterns are associated with AO+ (CP7, CP9 and CP10; Fig. 10c). These results indicate that the patterns in the bottom part of the SOM matrix are in general associated with AO+. Further, a larger positive AO in CP3, CP4, CP9 and CP12 during LWNT episodes in LIYs rather than those in HIYs suggests a link between the positive phase of the AO and the frequency of occurrence of episodes of enhanced LWNT during spring (see also Fig. 9). Note, however, that the AO index is based on pan-Arctic sea-level pressure anomalies and is not restricted to the investigation area. Hence, this association between the circulation patterns and the AO might only partly explain the processes behind the more frequent occurrence of LWNT episodes during LIYs over the investigation area.

## Discussion and conclusions

Five years that exhibit significant negative September SIC anomalies over the Laptev, East Siberian and Beaufort Seas during 1979–2012 are characterized by frequent and intense episodes of positive surface LWNT anomalies during spring, here defined as March 1 to melt onset. In contrast, the spring in years with anomalously high September SIC are characterized by fewer and shorter positive LWNT episodes (Table 1). Further, the timing of melt onset occurs significantly earlier for most of the LIYs (Table 2). This leads to a decrease of the surface albedo early in the season hereby enhancing the ice-albedo feedback, leading to an increased absorption of shortwave radiation throughout the rest of the melt season and hence to an accelerated ice melt (e.g., Kapsch et al. 2016).

To investigate the atmospheric circulation patterns associated with the springtime LWNT episodes, a SOM algorithm was applied to all spring days from 1979 to 2012. Two distinct atmospheric circulation patterns prevail: one similar to the Arctic Dipole anomaly, the other associated with the positive phase of the Arctic Oscillation. The AD-like patterns are characterized by low geopotential heights along the Russian coast and high geopotential heights along the Canadian coast. The AO+ patterns are associated with low geopotential heights over the majority of the Arctic, favoring transport from Siberia or the Kara Sea into the study region. CP1, CP3 and CP4 are the most frequent of the AD patterns, occurring over a third of the time in spring (15, 10 and 11% of the days, respectively; Fig. 5). The most frequent AO+ patterns are CP9, CP10 and CP12, prevailing more than another third of the spring days (10, 11 and 16%, respectively). During LWNT episodes in LIY springs, the AO+ patterns occur more frequently and AD patterns less frequently, compared to HIY-spring LWNT episodes.

During the LWNT episodes in spring of LIYs, the AO+ patterns are associated with advection from Siberia that that for most patterns favors anomalous transport of moisture, leading to ConLE being positive; ConDE anomalies are negative, likely due to low temperatures over Siberia until June (not shown). The positive anomalies of ConLE are associated with more water vapor and clouds in the study domain, leading to positive LWD anomalies. Increased LWD during spring is the primary cause for melt onset (Persson 2012; Mortin et al. 2016) and is likely an important factor for September sea–ice variability (Kapsch et al. 2016). During LIYs these patterns occur relatively early in spring of LIYs, as compared to HIYs, and are rather persistent (Fig. 8), supporting an early preconditioning and melt onset of sea ice. ConLE anomalies are negative only for CP8, indicating that other processes contribute to the positive LWNT anomalies under those circulation conditions. One such process may be an eastward propagation of cloud anomalies from the Nansen Basin and parts of the Barents and Kara Seas. Liu et al. (2007) argued that cloud anomalies propagate along the cyclone tracks that traverse from the North Atlantic along the Russian coast towards the East Siberian Sea. Specifically, CP9 and CP10 are characterized by cyclonic flow, supporting the notion of enhanced transport across the western boundary of the investigation area (Fig. 5). Note that mechanical wind forcing associated with sea–ice transport might also play a role; however, here we find no significant anomaly in the wind velocities for this specific AO+ pattern (not shown).

The AD patterns favor the advection of warm and moist air from the Pacific and adjacent seas into the investigation area during LIY LWNT episodes. Another mechanism associated with AD patterns that can contribute to negative September SICs over the investigation area is associated with sea–ice export and advection of heat within the ocean. Rigor et al. (2002) found that the AD patterns transport sea ice from the Siberian Arctic towards Canada, which leads to ice divergence in the central Arctic and therefore over the investigation area. Wang et al. (2009) and Maslanik et al. (2007) showed that AD favors sea–ice export from the Atlantic side of the Arctic, leading to an increased ocean heat flux into the Arctic Ocean through the Bering Strait. The LWNT episodes from 23 April to 19 May 1990 were associated exclusively with the patterns CP3, CP4, CP8 and CP10 and exhibit a SIC reduction after AD patterns had prevailed (Fig. 3). As all of these patterns are associated with positive anomalies of surface wind speeds over the investigation area (not shown), these might be additional factors that contribute to the negative ice anomalies.

Note that LWNT episodes occur not only in LIYs or HIYs. Throughout all the springs considered, 272 LWNT episodes occurred, spanning a total of 1156 days (Sect. 3.2; Table 1). Significant lagged correlations between LWNT episodes and anomalies of ConLE are evident in all years from 1979 to 2012, except 2010 (not shown). This indicates that the correlations between ConLE and episodes of enhanced LWNT are not unique to LIYs but, as would be expected, that enhanced moisture convergence always triggers LWNT anomalies. During spring of LIYs, however, LWNT episodes last considerably longer than average (Table 1), suggesting that atmospheric circulation patterns associated with enhanced moisture transport are more persistent in LIY springs.

Two questions were posed at the beginning of this paper. Regarding the first question—do similar atmospheric circulation patterns prevail in spring of all LIYs—the answer is inconclusive. We do find similarities in the atmospheric circulation in spring of LIYs, but several flow patterns emerge, and some important flow patterns emerge in both LIYs and HIYs. However, we find a possible relationship between circulation patterns in LIYs and the positive phase of the Arctic Oscillation, which might favor episodes of enhanced LWNT due to increased energy convergence over the investigation area. Note, however, that some patterns, such as CP1, CP6 or CP10, are associated with energy divergence throughout LWNT episodes in LIYs and HIYs (not shown), which leaves a substantial gray zone in which other processes, such as sea–ice dynamics and mechanical wind forcing or oceanic processes, may or may not play important roles for a given year. As to the second question—whether there are systematic differences in the characteristics of the atmospheric transport events during spring of LIYs and HIYs—the answer is definitely yes, particularly with respect to strength and duration of the LWNT events. Both intensity and persistence of thermodynamic forcing from the atmosphere in spring are larger for LIYs than for HIYs (Table 1). The significant difference in the persistence and timing of the individual flow patterns in spring prior to September LIYs and HIYs suggests that the distinct circulation patterns (CP9, CP10 and CP12) tend to occur earlier in spring and thus have longer time to act on the surface during LIYs (Figs. 8, 9; Table 1).

## Electronic supplementary material

Below is the link to the electronic supplementary material.

Supplementary material 1 (PDF 973 KB)

## References

[CR1] Burt MA, Randall DA, Branson MD (2016). Dark warming. J Clim.

[CR3] Comiso JC, Parkinson CL, Gersten R, Stock L (2008). Accelerated decline in the Arctic sea ice cover. Geophys Res Lett.

[CR4] Cox CJ, Uttal T, Long CN, Shupe MD, Stone RS, Starkweather S (2016). The role of springtime arctic clouds in determining autumn sea ice extent. J Clim.

[CR5] Dee DP, Uppala S (2009). Variational bias correction of satellite radiance data in the ERA-Interim reanalysis. QJR Meteorol Soc.

[CR6] Dee DP (2011). The ERA-Interim reanalysis: configuration and performance of the data assimilation system. QJR Meteorol Soc.

[CR7] Devasthale A, Sedlar J, Koenigk T, Fetzer EJ (2013). The thermodynamic state of the Arctic atmosphere observed by AIRS: comparisons during the record minimum sea ice extents of 2007 and 2012. Atmos Chem Phys.

[CR8] Ding Q (2017). Influence of high-latitude atmospheric circulation changes on summertime Arctic sea ice. Nat Clim Change.

[CR9] Döscher R, Vihma T, Maksimovich E (2014). Recent advances in understanding the arctic climate system state and change from a sea ice perspective: a review. Atmos Chem Phys Discuss.

[CR10] Eastman R, Warren SG (2010). Interannual variations of Arctic cloud types in relation to sea ice. J Clim.

[CR11] Graversen RG, Källén E, Tjernström M, Körnich H (2007). Atmospheric mass-transport inconsistencies in the ERA-40 reanalysis. Q J R Meteorol Soc.

[CR12] Graversen RG, Mauritsen T, Drijfhout S, Tjernström M, Mårtensson S (2011). Warm winds from the Pacific caused extensive Arctic sea–ice melt in summer 2007. Clim Dyn.

[CR13] Hewitson BC, Crane RG (2002). Self-organizing maps: applications to synoptic climatology. Clim Res.

[CR14] IPCC (2013), Climate change 2013: the physical science basis. Contribution of Working Group I to the Fifth Assessment Report of the Intergovernmental Panel on Climate Change. In: Stocker TF, Qin D, Plattner G-K, Tignor M, Allen SK, Boschung J, Nauels A, Xia Y, Bex V, Midgley PM (eds) Cambridge University Press, Cambridge, pp 1535. 10.1017/CBO9781107415324

[CR15] Jakobson E, Vihma T, Palo T, Jakobson L, Keernik H, Jaagus J (2012). Validation of atmospheric reanalyses over the central Arctic Ocean. Geophys Res Lett.

[CR16] Kapsch M-L, Graversen RG, Tjernström M (2013). Springtime atmospheric energy transport and the control of Arctic summer sea–ice extent. Nat Clim Change.

[CR17] Kapsch M-L, Graversen RG, Tjernström M, Bintanja R (2016). The effect of downwelling longwave and shortwave radiation on Arctic summer sea ice. J Clim.

[CR18] Kauker F, Kaminski T, Karcher M, Giering R, Gerdes R, Voßbeck M (2009). Adjoint analysis of the 2007 all time Arctic sea–ice minimum. Geophys Res Lett.

[CR19] Kay JE, Holland MM, Jahn A (2011). Inter-annual to multi-decadal Arctic sea ice extent trends in a warming world. Geophys Res Lett.

[CR20] Kohonen T (1982). Self-organized formation of topologically correct feature maps. Biol Cybern.

[CR21] Kohonen T (2001). Self-Organizing Maps, Third Edition.

[CR22] Lee HJ, Kwon MvO, Yeh S, Kwon Y, Park W, Park J, Kim YH, Alexander MA (2002). Impact of poleward moisture transport from the North Pacific on the acceleration of sea ice loss in the Arctic since. J Clim.

[CR23] Lindsay R, Wensnahan M, Schweiger A, Zhang J (2014). Evaluation of seven different atmospheric reanalysis products in the Arctic. J Clim.

[CR24] Liu Y, Key JR, Francis JA, Wang X (2007). Possible causes of decreasing cloud cover in the Arctic winter, 1982–2000. Geophys Res Lett.

[CR25] Maslanik JA, Fowler C, Stroeve J, Drobot S, Zwally J, Yi D, Emery W (2007). A younger, thinner Arctic ice cover: Increased potential for rapid, extensive sea–ice loss. Geophys Res Lett.

[CR27] Mortin J, Howell SEL, Wang L, Derksen C, Svensson G, Graversen RG (2014). Extending the QuikSCAT record of seasonal melt–freeze transitions over Arctic sea ice using ASCAT. Remote Sens Environ.

[CR28] Mortin J, Svensson G, Graversen RG, Kapsch M-L, Stroeve J, Boisvert LN (2016). Melt onset over Arctic sea ice controlled by atmospheric moisture transport. Geophys Res Lett.

[CR29] Ogi M, Wallace JM (2012). The role of summer surface wind anomalies in the summer Arctic sea ice extent in 2010 and 2011. Geophys Res Lett.

[CR30] Overland JE, Wang M (2005). The third Arctic climate pattern: 1930s and early 2000s. Geophys Res Lett.

[CR31] Overland JE, Wang M, Salo S (2008). The recent Arctic warm period. Tellus Ser A.

[CR32] Persson POG (2012). Onset and end of the summer melt season over sea ice: thermal structure and surface energy perspective from SHEBA. Clim Dyn.

[CR33] Reusch DB, Alley RB, Hewitson BC (2005). relative performance of self-organizing maps and principal component analysis in pattern extraction from synthetic climatological data. Polar Geogr.

[CR34] Rigor IG, Wallace JM (2002). Response of sea ice to the Arctic oscillation. J Clim.

[CR35] Serreze MC, Stroeve J (2015). Arctic sea ice trends, variability and implications for seasonal forecasting. Philis Trans R Soc.

[CR36] Sheridan SC, Lee CC (2011). The self-organizing map in synoptic climatological research. Prog Phys Geogr.

[CR37] Skific N, Francis JA (2012), Self-organizing maps: a powerful tool for the atmospheric sciences, applications of self-organizing maps. In: Johnsson M (ed) ISBN: 978-953-51-0862-7. InTech. 10.5772/54299

[CR38] Skific N, Francis JA, Cassano JJ (2009). Attribution of projected changes in atmospheric moisture transport in the Arctic: a self-organizing map perspective. J Clim.

[CR39] Stroeve JC, Markus T, Boisvert L, Miller J, Barrett A (2014). Changes in Arctic melt season and implications for sea ice loss. Geophys Res Lett.

[CR40] Thompson DWJ, Wallace JM (1998). The Arctic oscillation signature in the wintertime geopotential height and temperature fields. Geophys Res Lett.

[CR41] Trenberth KE (1991). Climate diagnostics from global analysis: Conservation of mass in ECMWF analysis. J Clim..

[CR44] Wang J, Zhang J, Watanabe E, Ikeda M, Mizobata K, Walsh JE, Bai X, Wu B (2009). Is the Dipole Anomaly a major driver to record lows in Arctic summer sea ice extent?. Geophys Res Lett.

[CR45] Wang C, Granskog MA, Hudson SR, Gerland S, Pavlov AK, Perovich DK, Nicolaus M (2016). Atmospheric conditions in the central Arctic Ocean through the melt seasons of 2012 and 2013: impact on surface conditions and solar energy deposition into the ice-ocean system. J Geophys Res Atmos.

[CR46] Zhang J, Lindsay R, Steele M, Schweiger A (2008). What drove the dramatic retreat of the arctic sea ice during summer 2007?. Geophys Res Lett.

