# Supplementary material for: Summers with low Arctic sea ice linked to persistence of spring atmospheric circulation patterns
Source: Clim Dyn. 2018 May 30;52(3):2497–512. doi: 10.1007/s00382-018-4279-z (PMC6413483; doi:10.1007/s00382-018-4279-z)
Supplement: Supplementary file 1 — Supplementary material 1 (PDF 973 KB) [file 382_2018_4279_MOESM1_ESM.pdf]

Summers with low Arctic sea ice linked to persistence of spring atmospheric circulation patterns

Supplementary Material
Climate Dynamics

Marie-Luise Kapsch*, Natasa Skific, Rune G. Graversen, Michael Tjernström, Jennifer A. Francis

***Corresponding author:** Marie-Luise Kapsch, Max Planck Institute for Meteorology, Bundesstraße 53, 20146 Hamburg, Germany.
e-mail: marie.kapsch@mpimet.mpg.de

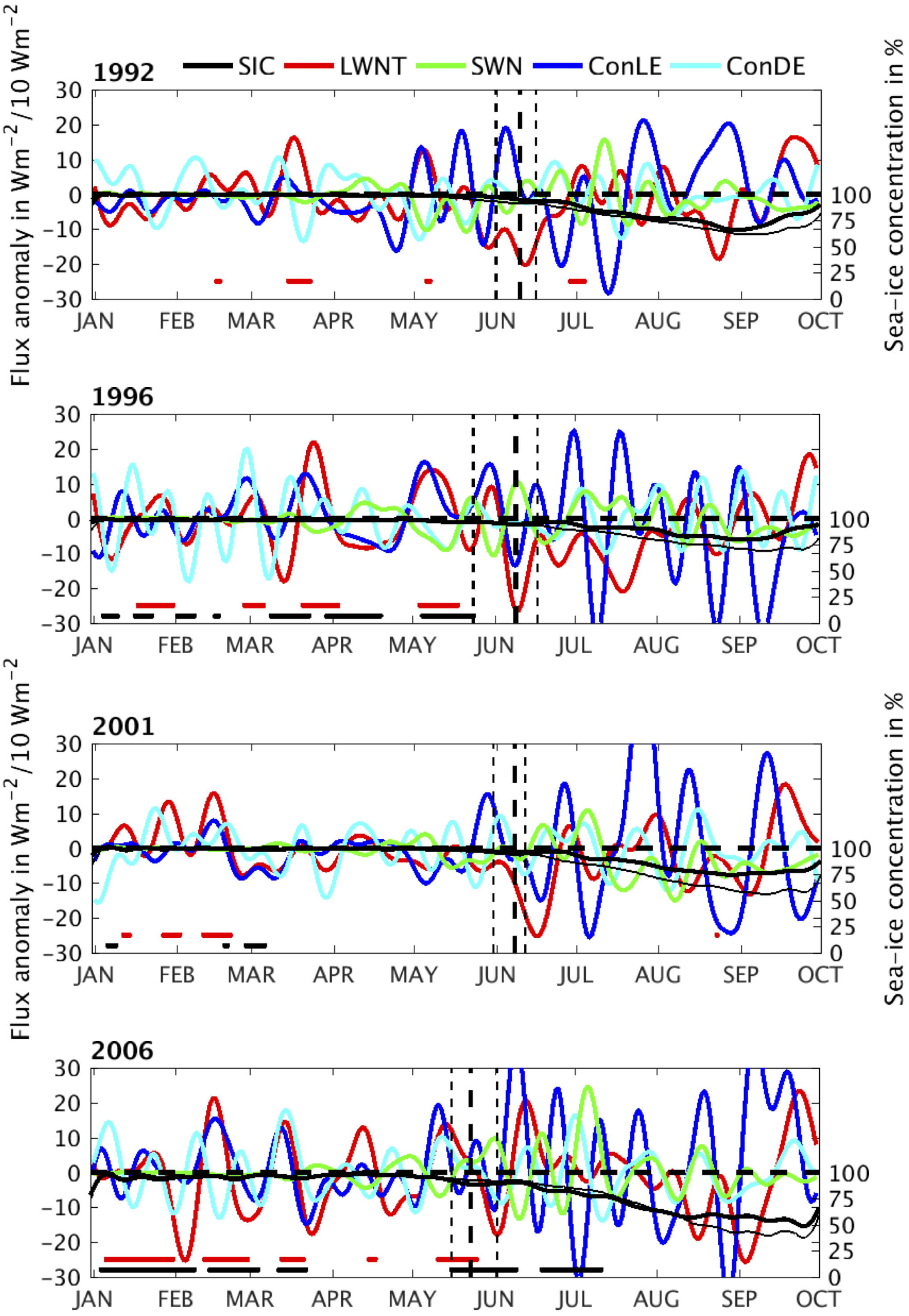

Fig. S1: Similar to Fig. 3 but for HIYs.

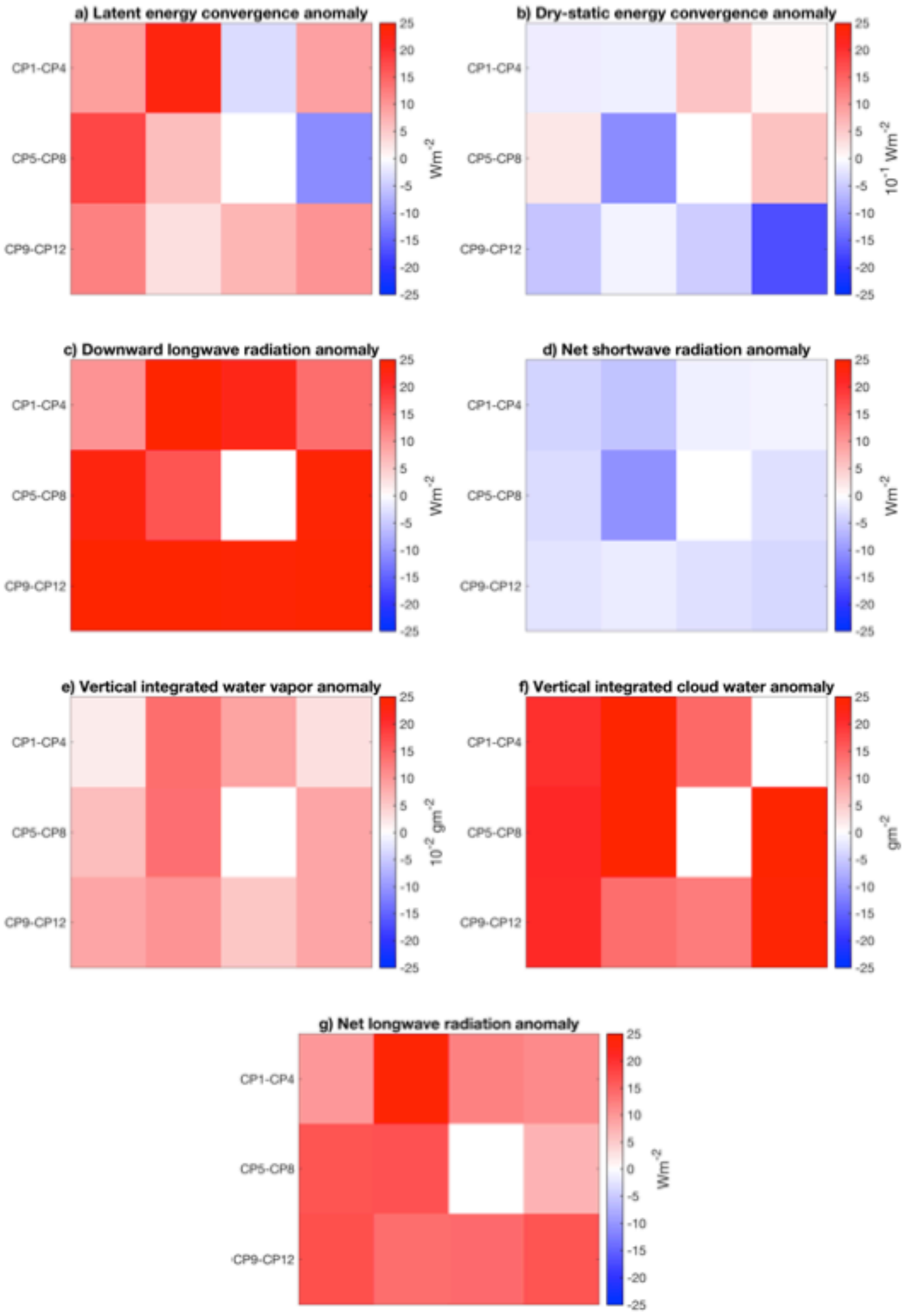

Fig S2: Similar to Fig. 7 but for the difference between spring LWNT episodes and the remaining spring days (RSDs) of LIYs.

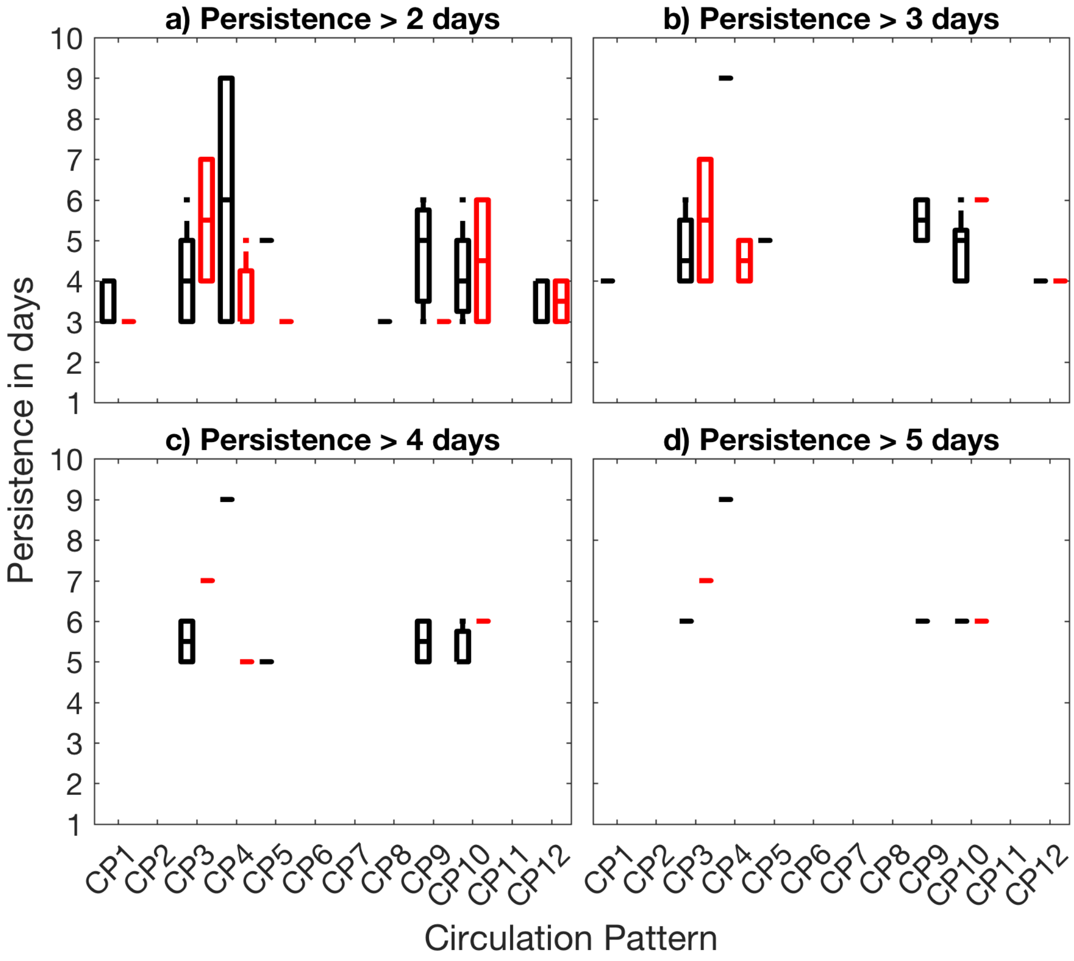

Fig. S3: Persistence of each pattern for LWNT episodes in LIYs and HIYs, as shown in Fig. 8 and 9. The barplots are created using events that lasted for a minimum of a) three, b) four, c) five or d) six consecutive days.
